# Prognostic factors in vagus nerve stimulation for drug-resistant epilepsy. Results from a systematic review and meta-analysis of the literature

**DOI:** 10.1007/s10143-026-04342-6

**Published:** 2026-06-17

**Authors:** Renata Martinelli, Gabriele Ciaffi, Filomena Fuggetta, Manuela D’Ercole, Benedetta Burattini, Alessandro Izzo, Nicola Montano

**Affiliations:** 1 Department of Neurosurgery, Fondazione Policlinico Universitario Agostino Gemelli IRCCS, largo A. Gemelli, 8, Rome, 00168 Italy; 2 Department of Neuroscience, Università Cattolica del Sacro Cuore, Largo F. Vito, 1, Rome, 00168 Italy

**Keywords:** Vagus nerve stimulation, Drug resistant epilepsy, VNS, Seizure outcome, Biomarkers, Prognostic factors

## Abstract

The aim of the present study was to conduct a systematic review and meta-analysis evaluating prognostic factors of response to treatment with VNS implantation in patients with drug-resistant epilepsy. We conducted a systematic review following the PRISMA 2020 guidelines to critically analyze relevant studies. The review question was formulated using the PICO framework: “In patients with drug-resistant epilepsy (P) undergoing VNS implantation (I) and subjected to preoperative and postoperative clinical and instrumental evaluations (C), can prognostic factors for therapeutic response (O) be identified?“. As outcome variables for metanalysis evaluation, gender, age at epilepsy onset, age at VNS implantation, focal onset of seizures, epilepsy duration and genetic etiology were evaluated. The protocol for this systematic review and meta-analysis was registered in the PROSPERO database (registration number: CRD420261333961). The literature search yielded a total of 900 results. After removing duplicates, 571 papers were screened. Ultimately 39 were deemed relevant. There was a statistically significant association between focal seizures and VNS response (RR 1.31, 95% CI 1.02–1.69, *p* < 0.05), gender (RR 1.14, 95% CI 1.02–1.26, *p* < 0.05) and younger age at seizure onset (SMD 0.22, 95% CI 0.02–0.41, *p* = 0.028), suggesting a slightly higher probability of response in these subgroups. No significant associations were found for genetic etiology, epilepsy duration, or age at VNS implantation. Heterogeneity was generally low across analyses, except for focal seizures (I2 = 49%). Focal seizures and younger age at epilepsy onset are associated with improved response to VNS therapy. These findings support the role of early patient stratification and suggest that VNS should be considered earlier in selected patients. While several promising biomarkers have been identified, further research is needed to establish their clinical utility and develop more accurate patient selection frameworks.

## Introduction

Around 30% of epilepsy patients continue to experience seizures despite individualized medical treatment [1]. While surgical resection of the epileptic focus can be curative for selected patients, many are unsuitable candidates or continue to experience seizures even after undergoing resective surgery [2].

Vagus nerve stimulation (VNS) was first approved in 1997 as a treatment for patients with medically refractory seizures, after Zabara hypothesized that VNS could reduce epileptic seizures by desynchronizing electroencephalographic (EEG) activity through neurotransmitter modulation [3].

Long-term studies subsequently demonstrated sustained seizure reductions [4, 5] and improvements in quality of life in both adult and pediatric populations [6, 7] establishing VNS as a viable option for reducing seizure frequency and improving daily living tasks in patients with intractable epilepsy [4, 8, 9, 10]. Recent studies have increasingly focused on the long-term effectiveness of VNS. However, despite its established role, prognostic factors that could guide early implantation and identify patients most likely to benefit remain poorly defined. The aim of this systematic review and meta-analysis is to identify prognostic factors associated with VNS response in patients with drug-resistant epilepsy (DRE).

## Materials and methods

### Systematic review

**Fig. 1:**
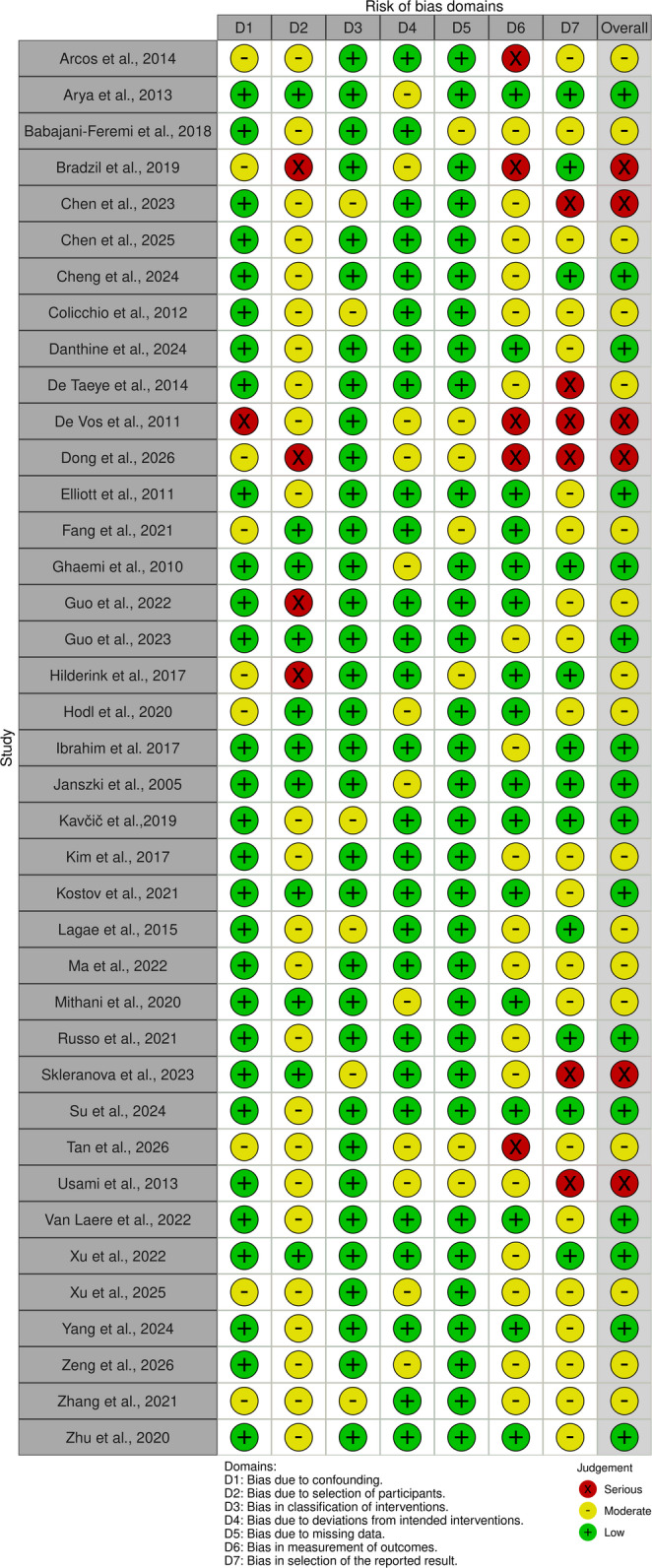
ROBINS-I assessment tool

This systematic review adhered to PRISMA 2020 guidelines, for identifying and critically evaluating relevant studies. All procedures followed the protocols outlined in the Cochrane Handbook of Systematic Reviews and Meta-analysis of Interventions (version 6.3) [11, 12]. The review question was structured using the PICO framework: “In patients with DRE (P) undergoing VNS implantation (I) and subjected to preoperative and postoperative clinical and instrumental evaluations (C), can prognostic factors for therapeutic response (O) be identified?“ [13]. Pubmed and Scopus were searched on the 3rd of January 2026 using comprehensive search terms: “(“vagus nerve stimulation” OR “VNS”) AND (“refractory epilepsy” OR “drug-resistant epilepsy”) AND (“predictors of outcome” OR “prognostic factors” OR “response predictors” OR “efficacy”)”.Two authors (R.M. and G.C.) independently conducted the abstract screening for eligibility. Any discordance was solved by consensus with a third, senior author (N.M.). We included studies evaluating prognostic factors of outcome after VNS in patients with DRE. Excluded were non-English studies, reviews, case reports, non-human studies, letters, and studies assessing only VNS efficacy. In all the evaluated studies, patients were considered responders to VNS if a reduction > 50% of seizures was obtained after undergoing VNS, according to McHugh classification [14]. Subgroup analyses according to epilepsy syndrome, age category, and follow-up duration were planned at protocol stage; however, owing to inconsistent reporting and heterogeneous classification across the included studies, formal quantitative subgroup analyses were not feasible.The risk of bias was assessed using the ROBINS-I assessment tool [15] (see Fig. 1). The protocol for this study was prospectively registered in the PROSPERO database (registration number: CRD420261333961).

### Meta-analysis and statistical analysis

Variables were eligible for quantitative meta-analysis if they were reported in at least three independent studies, they were operationally defined in a comparable way across studies, and sufficient quantitative data were available to allow effect-size estimation. Therefore gender, age at epilepsy onset, age at VNS implantation, focal onset of seizures, epilepsy duration and genetic etiology were evaluated as outcomes variables. Imaging-derived variables and most electrophysiological measures did not satisfy these criteria owing to substantial heterogeneity in acquisition protocols and outcome metrics, and were therefore synthesized qualitatively only. Statistical analyses were performed using MetaAnalysisOnline.com [15]. Heterogeneity was tested using the c2 test and quantified by calculating the I2 statistic, in which *P* < 0.05 and I2 > 50% were considered statistically significant. Dichotomous variables were analyzed using risk ratios (RR), while continuous variables were analyzed using standardized mean differences (SMD) to account for variability in measurement scales across studies. A random-effects model was applied in all analyses. Publication bias was tested using the funnel plot.

## Results

### Systematic review

**Fig. 2:**
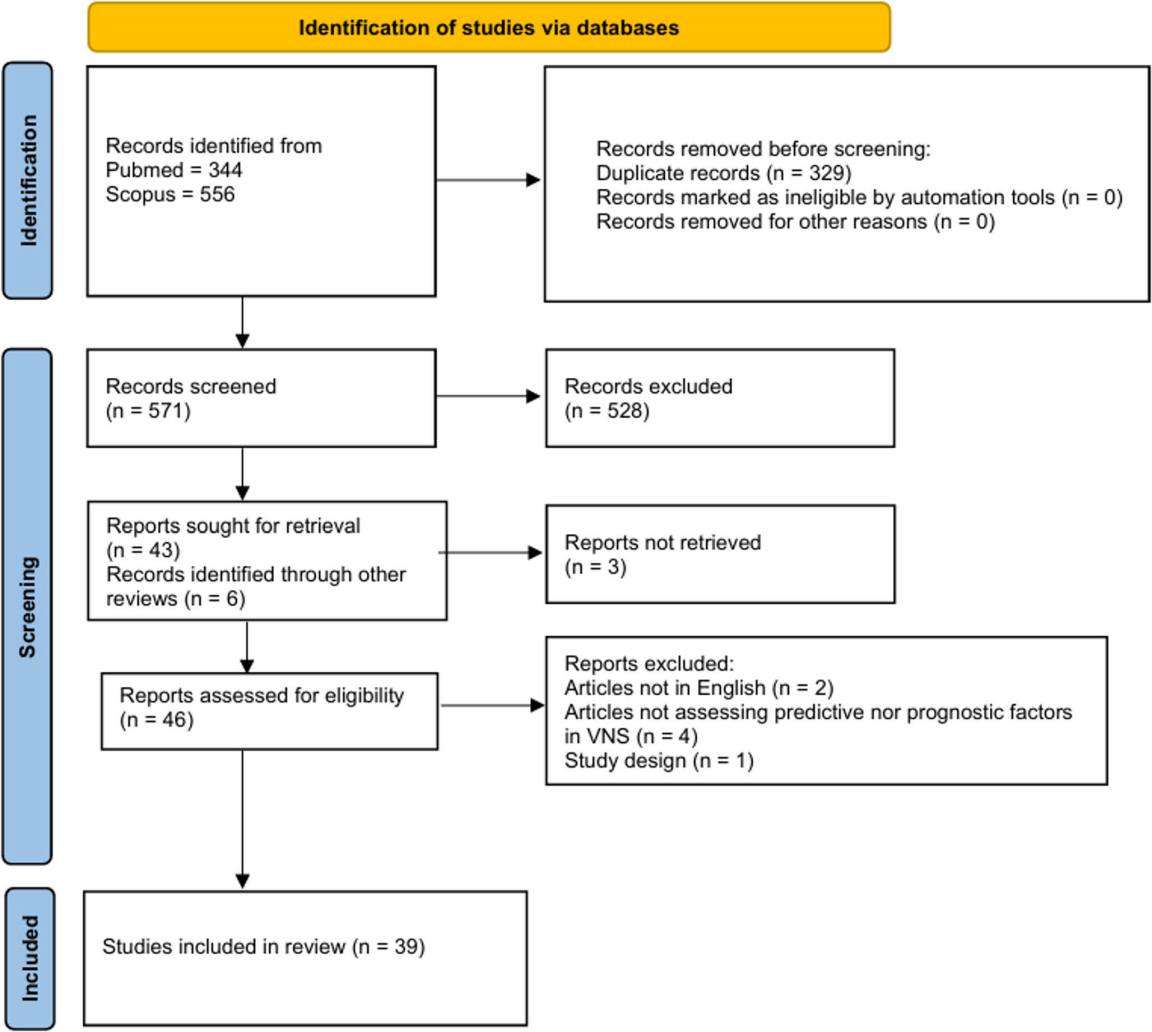
PRISMA flow chart

The search of the literature yielded a total of 900 results. After removal of duplicates (*n* = 329), 571 papers were screened, and 528 records were excluded via title and abstract screening. After full text examination, 39 studies were included in this review (see Fig. 2), including 8 prospective and 31 retrospective studies (see Table 1). A qualitative evaluation lead to identification of EEG markers, clinical factors, predictive models, and other emerging biomarkers of outcome prediction in DRE patients undergoing VNS implantation.

**Table 1:** Studies included in this systematic review

Author, year	Type of study	Type of marker
Arcos et al., 2014 [18]	retrospective	EEG
Arya et al., 2013 [48]	retrospective	Predictive model
Babajani-Feremi et al., 2018 [23]	retrospective	EEG
Bradzil et al., 2019 [45]	retrospective	Predictive model
Chen et al., 2023 [47]	retrospective	Predictive model
Chen et al., 2025 [35]	retrospective	Clinical
Cheng et al., 2024 [42]	retrospective	Predictive model
Colicchio et al., 2012 [27]	prospective	Clinical
Danthine et al., 2024 [44]	retrospective	Predictive model
De Taeye et al., 2014 [26]	retrospective	EEG
De Vos et al., 2011 [19]	retrospective	EEG
Dong et al., 2025 [34]	retrospective	Clinical
Elliott et al., 2011 [9]	retrospective	EEG
Fang et al., 2021 [55]	retrospective	Clinical
Ghaemi et al., 2010 [39]	retrospective	Predictive model
Guo et al., 2022 [16]	retrospective	EEG
Guo et al., 2023 [49]	retrospective	Imaging
Hilderink et al., 2017 [20]	prospective	EEG
Hodi et al., 2020 [38]	Prospective	Clinical
Ibrahim et al. 2017 [24]	prospective	Predictive model
Janszky et al., 2004 [40]	retrospective	Predictive model
Kavčič et al., 2019 [28]	retrospective	Clinical
Kim et al., 2017 [17]	retrospective	EEG
Kostov et al., 2021 [37]	retrospective	Clinical
Lagae et al., 2015 [29]	retrospective	Clinical
Ma et al., 2022 [43]	retrospective	Predictive model
Mithani et al., 2020 [54]	retrospective	Clinical
Russo et al., 2021 [30]	retrospective	Clinical
Sklenarova et al., 2023 [21]	retrospective	EEG
Su et al., 2024 [46]	retrospective	Predictive model
Tan et al., 2025 [32]	retrospective	Clinical
Usami et al., 2013 [53]	prospective	IONM
Van Laere et al., 2022 [50]	retrospective	Imaging
Xu et al., 2022 [31]	retrospective	Clinical
Xu et al., 2025 [51]	prospective	Imaging
Yang et al., 2024 [22]	prospective	EEG
Zeng et al., 2026 [36]	Prospective	Clinical
Zhang et al., 2021 [52]	retrospective	Genetic
Zhu et al., 2020 [33]	retrospective	Clinical

### EEG markers

Across 11 studies electrophysiological parameters were extensively investigated (see Table 2). Despite methodological heterogeneity in acquisition protocols and analytic techniques, some common patterns could be identified. A consistent finding concerns patients presenting with focal or multifocal interictal epileptic discharges (IEDs), who demonstrated higher response rates compared with those exhibiting generalized epileptiform activity [16, 17]. Similarly, focal EEG abnormalities and temporal lobe discharges were associated with improved seizure control and earlier response [9, 18], whereas non responders (NR) more frequently exhibited bilateral or generalized patterns [19, 20]. The prognostic value of specific oscillatory bands was also highlighted by the analyses of frequency domains. The alpha band emerged as particularly informative in distinguishing R from NR patients [21], while alterations in theta and beta activity also showed discriminatory capacity [22, 23]. More advanced approaches based on EEG or MEG, reinforced the hypothesis that VNS response is related to large-scale network organization [24, 25]. MEG-based graph measures, including modularity and connectivity patterns, were found to differ significantly between R and NR [23]. Measures of hemispheric symmetry further demonstrated that increased interhemispheric asymmetry is associated with poorer outcomes [19, 20] and noradrenergic system engagement expressed by P3 amplitude modulation, was proposed as noninvasive indicator of VNS efficacy [26] supporting the mechanistic link between locus coeruleus activation and therapeutic response. Overall evidence shows that patients with focal epileptiform patterns, preserved network organization, and specific oscillatory characteristics are more likely to benefit from VNS therapy.

**Table 2:** Studies evaluating EEG markers

Author, year	N. of patients	Sex	Mean age atimplantation	Epilepsy duration before VNS (years)	Seizure control (responders, non responders)	Specific Marker	Outcomes
Yang et al., 2024[22]	18	F = 13, M = 5	14.53	5.25	*R* = 10 NR = 8	EEG aperiodic parameters	EEG aperiodic parameters are promising biomarkers for VNS efficacy.
Sklenarova et al., 2023[21]	59	F = 34, M = 25	33	20.5	*R* = 24 NR = 35	Entropy models	R and NR were well differentiated by spectral entropy EEG segments.
Guo et al., 2022[16]	45	F = 10, M = 35	22.2	4	*R* = 29 NR = 16	IEDs	Patients with focal or multifocal IEDs were found to have better efficacy after VNS therapy.
Babajani-Feremi et al., 2018[23]	25	F = 13, M = 12	11.3 ± 6.9	6.25	*R* = 14 NR = 9	rs-MEG data	MEG-based graph measures are reliable biomarkers to predict seizure outcome of VNS treatment.
Kim et al., 2017[17]	58	F = 17, M = 41	10.9	NA	*R* = 29 NR = 30	IEDs	Patients with focal or multifocal epileptic form discharges were more likely to be responders.
Arcos et al., 2014[18]	37	F = 2, M = 16	36.03 ± 14.91	25.78 ± 14.04	*R* = 23 NR = 14	video-EEG	Temporal lobe discharges are indicators of an early response; MRI lesions indicate a late response to VNS.
De Taeye et al., 2014[26]	20	F = 12, M = 8	44	9	*R* = 10 NR = 10	P3 component of the event-related potential	Modulation of the P3 amplitude could be a noninvasive biomarker for the therapeutic efficacy of VNS.
Elliott et al., 2011[9]	400	F = 220, M = 216	29.0 ± 16.5	19.2 ± 13.0	*R* = 351 NR = 49	Focal EEG findings	Focal EEG findings predicted improved seizure control especially in case of exclusively focal seizures.
De Vos et al., 2011[19]	19	F = 8 M = 11	NA	NA	*R* = 9 NR = 10	pdBSI	quantifying EEG symmetry using the pdBSI shows promising results in predicting the reduction of seizure frequency after VNS treatment.
Janszky et al., 2004 [40]	47	F = 25 M = 22	22.7	17.8	*R* = 6 NR = 41		Absence of bilateral IEDs and presence of MCD showed a significant association with the seizure-free outcome.
Hilderink et al., 2017[20]	39	F = 24 M = 15	NA	NA	*R* = 10 NR = 29	pdBSI	pdBSI could predict which patients will benefit from VNS treatment. NR show significantly higher EEG asymmetry, reflected in higher pdBSI values, as compared with responders.

*F* female, *M* male, *R* responder, *NR* non responder, *VNS* vagus nerve stimulation, *PTE* post traumatic epilepsy, *PSE* post stroke epilepsy, *IEDs* interictal epileptiform discharges

### Clinical factors

Clinical variables were investigated as prognostic factors of VNS response in 13 studies (see Table 3). Earlier intervention appeared to be one of the most reproducible indicator of favorable outcome, and younger age at implantation and shorter epilepsy duration were repeatedly associated with improved seizure control. Colicchio et al. [27] reported that patients with lesional etiology and shorter epilepsy duration undergoing implantation before 18 years of age showed superior outcomes, as confirmed by Kavčič et al. [28]and Lagae et al. [29], while Russo et al. [30] demonstrated that early implantation is independently associated with better long-term seizure reduction.

**Table 3:** Studies evaluating clinical markers

Author, year	*N*. of patients	Sex	Mean age at implantation	Epilepsy duration before VNS (years)	Seizure control (responders, non responders)	Outcomes
Xu et al., 2022 [51]	76	F = 27, M = 59	21.40 ± 9.09	13.12 ± 7.86	*R* = 52 NR = 24	The stimulation efficacy increases over time; early VNS implantation improves the prognosis.
Kostov et al., 2021 [37]	436	F = 213, M = 249	19.5	13	*R* = 257 NR = 179	Partial early response was a positive predictor of becoming a responder at 2 years. Unchanged therapy, PTE and PSE were associated to better responder rates.
Russo et al., 2021 [30]	27	NA	10.3	NA	*R* = 17 NR = 10	Early VNS implantation was a predictor of favorable outcome in pediatric patients with epileptic encephalopathy.
Zhu et al., 2020 [33]	77	F = 28, M = 49	18 ± 9.3	11.4 ± 7.7	*R* = 39 NR = 38	Epilepsy duration is an independent predictor for responders to VNS, especially in patients with a duration of 5–12.5 years.
Kavčič et al.,2019 [28]	48	F = 18, M = 21	18.1 ± 14.2	13.4 ± 11.3	*R* = 18 NR = 30	Implantation at a younger age and shorter duration of epilepsy could be important predictors of better outcome.
Lagae et al., 2015 [29]	70	NA	8	3.5, 11.5	*R* = 38 NR = 32	Younger age at VNS implantation might result in a better outcome.
Colicchio et al., 2012 [27]	55	F = 20, M = 33	30.67 ± 13.82	22.28 ± 10.49	*R* = 21 NR = 34	Patients with lesional etiology and a short duration of epilepsy, younger than 18 years, are the best candidates for VNS.
Hödl et al., 2020 [38]	30	F = 14 M = 16	39.57	NA	*R* = 15 NR = 15	VNS responders were significantly associated to lower P3b amplitude, lower HF power and more NREM 3.
Ghaemi et al., 2010 [39]	144	F = 77 M = 67	23.7 ± 13.4	17.4 ± 10.6	*R* = 10 NR = 134	Some forms of cortical dysgenesis may be associated to seizure-freedom after VNS, especially in patients with unilateral IEDs and earlier implantation.
Chen et al., 2025 [35]	65	F = 25 M = 40	9.8	6.0	*R* = 40 NR = 25	Age > 6 at seizure onset, seizures frequency < 30/month, focal seizures and epileptic encephalopathies are significant PF
Dong et al., 2025 [34]	44	F = 14 M = 30	18.47	8.45	*R* = 21 NR = 23	Longer preoperative epilepsy duration emerged as a significant negative predictor of treatment response. Only 2 patients achieved seizure-free status
Tan et al., 2025 [32]	99	F = 28 M = 71	15.72	8.77	*R* = 33 NR = 66	Multivariate regression analysis showed that structural etiology was a protective factor for effectiveness of VNS
Zeng et al., 2026 [36]	54	F = 26 M = 28	7.8	48	*R* = 32 NR = 22	VNS efficacy increased markedly over time. Significant improvements were observed in neurocognitive development and quality of life.

*F* female, *M* male, *R* responder, *NR* non responder, *VNS* vagus nerve stimulation, *PTE* post traumatic epilepsy, *PSE* post stroke epilepsy, *HF* high frequency, *IEDs* interictal epileptiform discharges, *MCD* malformation of cortical development

Four studies [31–34] reported a progressive increase in responder rates over time following implantation, whereas gender and chronological age alone did not demonstrate a significant impact on treatment efficacy. Seizure semiology also emerged as a relevant prognostic factor with focal seizures being independently associated with treatment response [35] whereas absence seizures, structural etiologies, and abnormal neuroimaging were associated with poorer outcomes in pediatric cohorts [32, 36]. Importantly, early treatment response appears to carry prognostic value, as Kostov et al. [37] demonstrated that achieving a partial response at six months is associated to a ≥ 75% seizure reduction at two years.

Additional physiological markers [38, 39] and the absence of bilateral interictal epileptiform discharges were also associated with improved outcomes [40]. Overall, earlier implantation, shorter epilepsy duration, and focal seizure type emerged as the most consistent prognostic factors of VNS response, according to this systematic review [41].

### Predictive models

Eight retrospective studies developed predictive models for VNS response using clinical and electrophysiological data (Table 4). Several models relied on EEG-derived metrics, including alpha and theta band activity [42], phase lag index connectivity measures [43, 44], and EEG reactivity differences [45]. Other approaches integrated clinical variables or functional connectivity patterns. Su et al. [46] used logistic regression of clinical data in pediatric patients, while Chen et al. [47] constructed models based on brain network connectivity. Consistently, enhanced thalamocortical connectivity was associated with favorable outcomes [24], and shorter epilepsy duration predicted better response in nonlesional patients [48]. Overall, these findings support the concept that VNS responsiveness reflects a multidimensional network-level phenomenon requiring further prospective validation.

**Table 4:** Studies assessing predictive models

Author, year	*N*. of patients	Sex	Mean age at implantation	Epilepsy duration before VNS (years)	Seizure control (responders, non responders)	Outcomes
Cheng et al., 2024 [42]	65	F = 29, M = 36	1.78–15.35	3.52	*R* = 38 NR = 27	Compared to NR, VNS responders had a more efficient α band brain network and less spectral complexity of θ brain activities.
Danthine et al., 2024 [44]	38	F = 20, M = 18	(18–75)	12.3	*R* = 12 NR = 26	Higher seizure reduction is correlated with a lower α band connectivity in sleep. NR patients have a greater functional connectivity in the δ band during wakefulness.
Su et al., 2024 [46]	45	F = 14, M = 31	8.93 ± 4.30	5.50	*R* = 25 NR = 20	Shorter seizure duration, focal seizure, and absence of intellectual disability were used to develop a nomogram for predicting the efficacy of VNS in children.
Chen et al., 2023 [47]	23	F = 6, M = 17	NA	3	*R* = 15 NR = 8	The postcentral gyrus was identified as the most significant differential brain region between VNS responders and NR.
Ma et al., 2022 [43]	70	F = 34, M = 54	5.6	3.3	*R* = 37 NR = 33	The average interictal awake PLI in the high β band was significantly higher in responders than NR.
Arya et al., 2013 [48]	43	F = 20 M = 23	10.9 ± 5.3	6.0 ± 4.9	*R* = 30 NR = 13	Nonlesional patients are significantly more likely to have better outcome with VNS.
Bradzil et al., 2019 [45]	60	F = 34, M = 26	33	22	*R* = 35 NR = 25	Power spectral analyses revealed the dynamics of α and γ activity significantly reflected VNS efficacy in R compared to NR.
Ibrahim et al. 2017 [24]	21	NA	(5–21)	NA	*R* = 11 NR = 10	Enhanced connectivity within thalamocortical circuitry, was found to be associated with seizure response to VNS.

*F* female, *M* male, *R* responder, *NR* non responder, *VNS* vagus nerve stimulation, *DRE* drug resistant epilepsy, *PLI* phase lag index

### Other emerging factors

Different emerging factors were evaluated in 7 studies (Table 5), many focusing on brain network organization and structural connectivity. Guo et al. [49] introduced the concept of brain clinical signatures, integrating imaging and clinical variables to assess VNS responsiveness. Van Laere et al. [50] reported that specific perfusion changes on SPECT imaging correlate with long-term therapeutic response. Structural connectivity analyses also supported a network-based mechanism: Xu et al. [51] demonstrated widespread reductions in thalamocortical fractional anisotropy in patients with DRE, while higher preoperative values in thalamocortical pathways were associated with better VNS response. Genetic susceptibility has also been explored and Zhang et al. [52] suggested a role for adenosine metabolism in treatment response. Electrophysiological markers of vagal conduction were proposed by Usami et al. [53], while Mithani et al. [54] showed that responders exhibit broader somatosensory evoked field localization and stronger functional connectivity on MEG. Finally, Fang et al. [55] reported that specific heart rate variability patterns during sleep may be prognostic factors of VNS response.

**Table 5:** Studies evaluating other emerging biomarkers

Author, year	*N*. of patients	Sex	Mean age at implantation	Epilepsy duration before VNS (years)	Seizure control (responders, non responders)	Specific marker	Outcomes
Guo et al., 2023 [49]	44	F = 60, M = 29	14.6	8.39	*R* = 22 NR = 44	Brain clinical signature	Regression analysis showed prediction potential of brain-clinical signature for VNS response.
Van Laere et al., 2022 [50]	23	F = 13, M = 11	32.4 ± 10.6	21 ± 11.7	*R* = 23 NR = 14	Perfusion SPECT changes	Acute amygdala and chronic hippocampal perfusion changes are predictive of long term response.
Zhang et al., 2021 [52]	194	F = 67, M = 127	16.38 ± 10.44	NA	*R* = 108 NR = 86	ADK SNPs	Three homozygous ADK SNPs may serve as useful biomarkers for prediction of VNS therapy outcome.
Usami et al., 2013 [53]	25	F = 10, M = 15	18	NA	*R* = 9 NR = 16	Scalp-Recorded Evoked Potentials	Recording of VN-EP might document the cause of treatment failure in some patients.
Mithani et al., 2020 [54]	12	F = 8 M = 2	*R* = 9.2 ± 6.6 NR = 13.5 ± 10.3	*R* = 4.9 ± 2.5 NR = 7.4 ± 4.7	*R* = 6 NR = 6	Median nerve somatosensory evoked fields	R had more widespread SEFs localization and greater functional connectivity within limbic and sensorimotor networks in response to MEG.
Fang et al., 2021 [55]	59	F = 19 M = 40	*R* = 19.6 ± 7.9 NR = 18.8 ± 8.3	*R* = 12.4 ± 7.4 NR = 10.6 ± 7.1	*R* = 30 NR = 29	Heart Rate Variability during sleep	Preoperative study of HRV during sleep could achieve a better performance of VNS outcome prediction.
Xu et al., 2025 [21]	31 30	F = 12, M = 19 F= 14 M = 16	DRE = 25.71 ± 14.65 HG = 26.53 ± 9.67	9.419 ± 7.089	*R* = 13 NR = 18	DTI for thalamo-cortical pathways	DRE patients with seizure frequency reduction on 6-months FU showed relatively intact thalamo-cortical connections. DTI data potentially predict the efficacy of VNS treatment

*F* female, *M* male, *R* responder, *NR* non responder, *VNS* vagus nerve stimulation, *SNPs* single nucleotide polymorphisms, *EP* evoked potentials, *SEFs* somatosensory evoked fields, *HRV* heart rate variability, *MEG* magnetoencephalography, *DRE* drug-resistent epilepsy (group), *HG* Healthy control group

### Metanalysis

In this meta-analysis we evaluated gender, age at epilepsy onset, age at VNS implantation, focal onset of seizures, epilepsy duration and genetic etiology as outcome variables.

#### Gender (male)

**Fig. 3:**
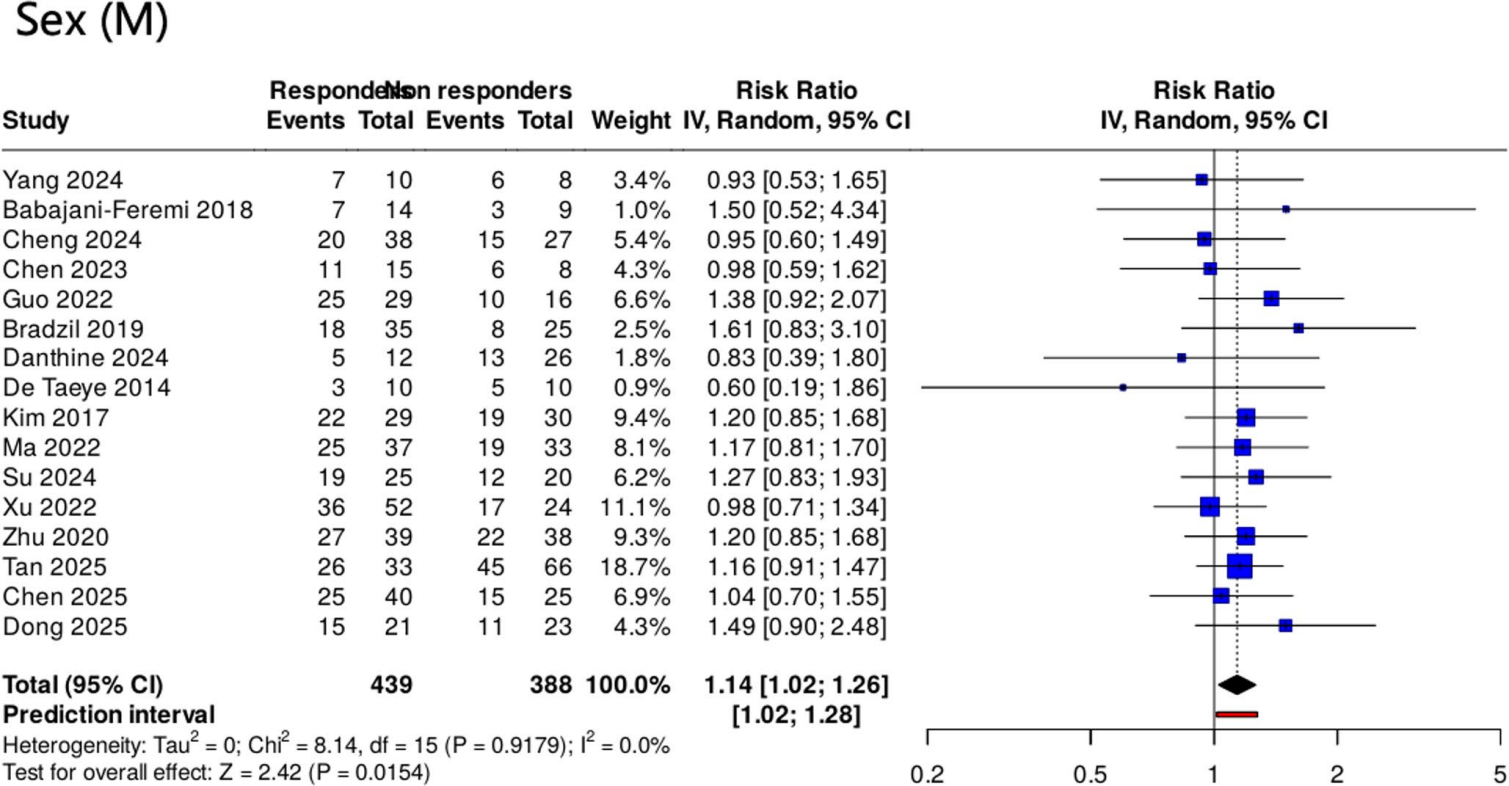
Gender forest plot

**Fig. 4:**
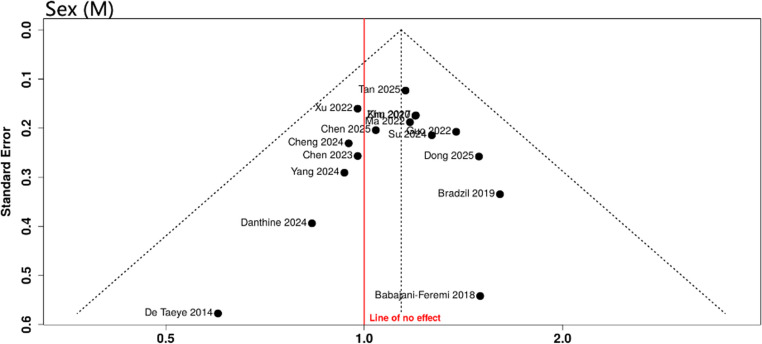
Gender funnel plot

All together 16 studies were analyzed with a total of 439 R and 388 NR. Based on the analysis performed using random effects model with inverse variance method to compare the RR, there is a statistical difference between the two cohorts, the overall risk ratio is 1.14 with a 95% CI of 1.02–1.26, indicating a higher proportion of male patients in the R cohort and therefore a small but statistically significant association of male sex with VNS response. The test for overall effect shows a significance at *p* < 0.05. We did not observe significant heterogeneity, suggesting that the effect sizes across studies were consistent in both magnitude and direction (Fig. 3). The funnel plot (Fig. 4) and Egger tests did not suggest publication bias. 

#### Age at onset of epilepsy

**Fig. 5:**
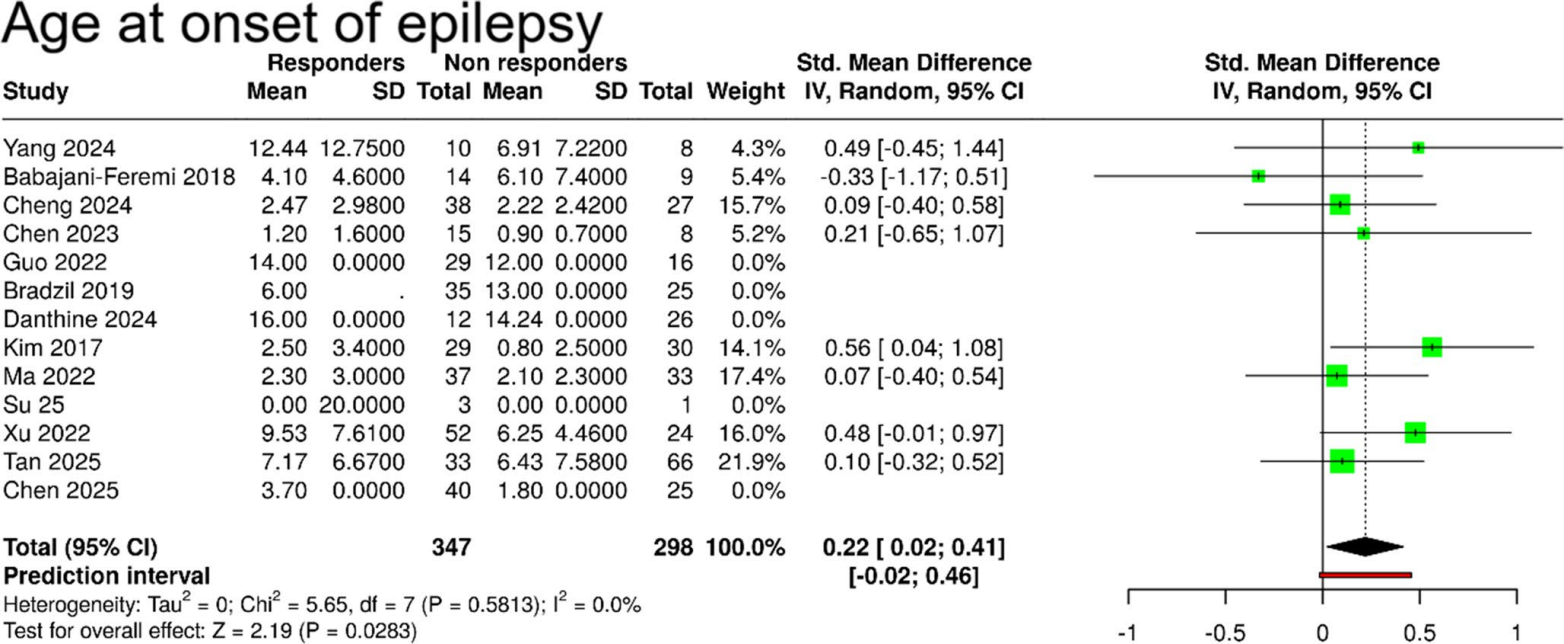
Age at onset of epilepsy forest plot

**Fig. 6:**
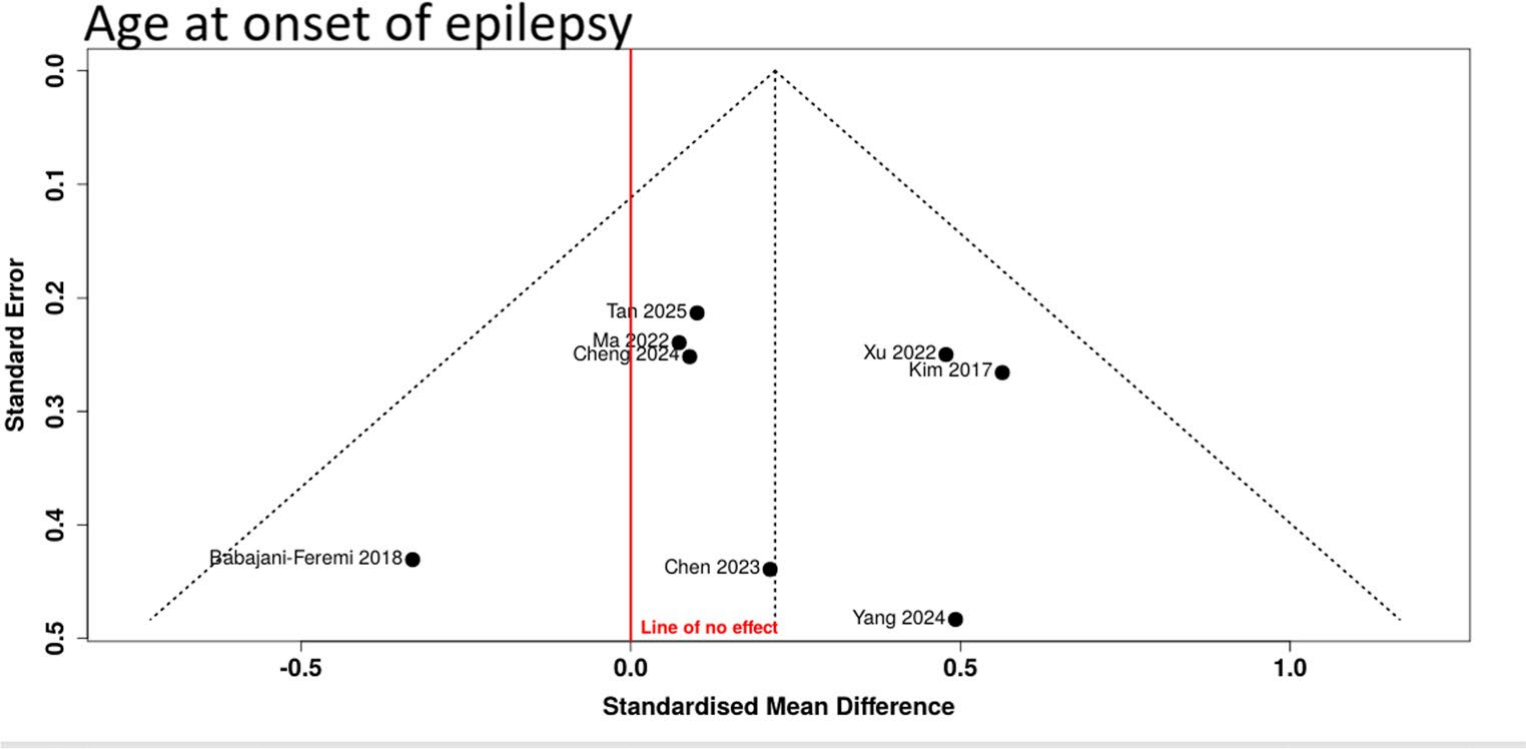
Age at onset of epilepsy funnel plot

Thirteen studies were included, comprising a total of 347 R and 298 NR. The analysis was performed using a random-effects model as previously described, and a significant difference was observed between R and NR with an overall standardized mean difference (SMD) of 0.22 (95% CI 0.02–0.41), indicating a younger age at seizure onset in R. The test for overall effect was statistically significant (Z = 2.19, *P* = 0.028). No significant heterogeneity was detected across studies (Fig. 5). The funnel plot (Fig. 6) and Egger tests did not suggest publication bias.

#### Genetic etiology of epilepsy

**Fig. 7:**
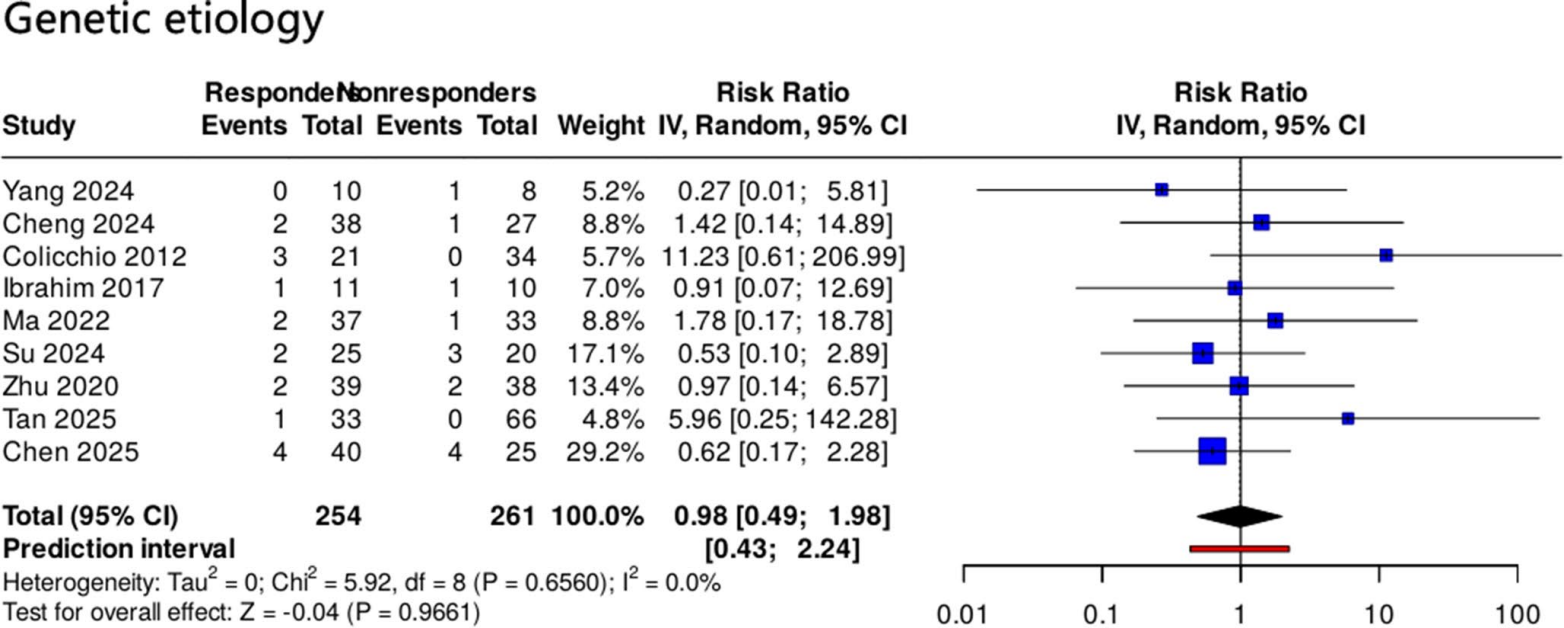
Genetic etiology of epilepsy forest plot

**Fig. 8:**
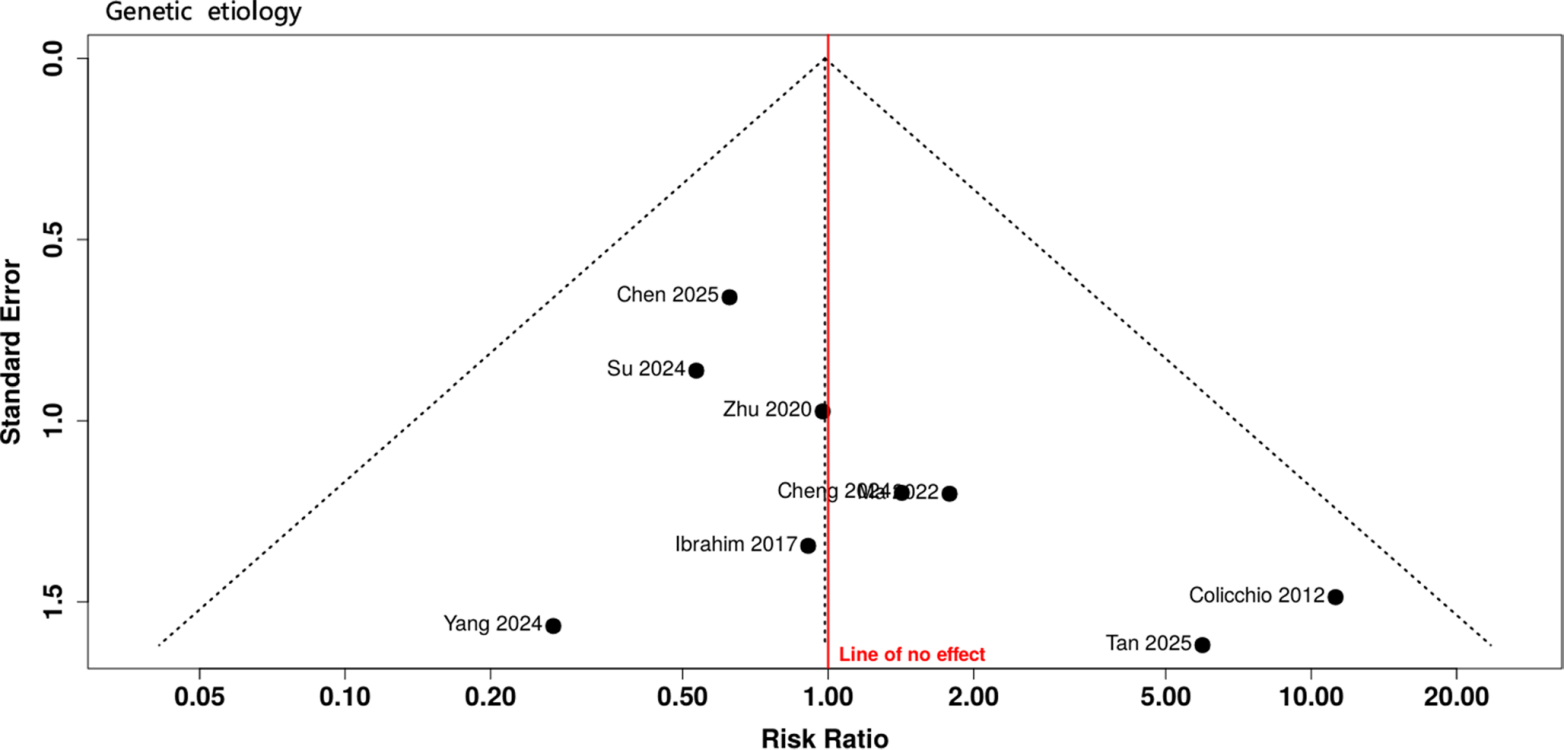
Genetic etiology of epilepsy funnel plot

Nine studies were included with a total of 254 subjects in the R cohort and 261 subjects in the NR cohort, with no statistical difference found between the two cohort, and an overall RR of 0.98 with a 95% CI of 0.49–1.98. The test for overall effect does not show a significant effect. No significant heterogeneity was observed (Fig. 7). The funnel plot and Egger tests did not suggest publication bias. (Fig. 8).

#### Epilepsy duration

**Fig. 9:**
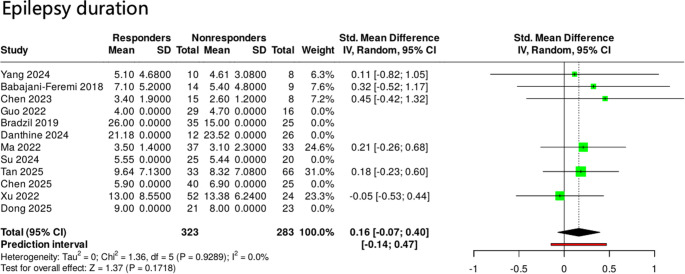
Epilepsy duration forest plot

**Fig. 10:**
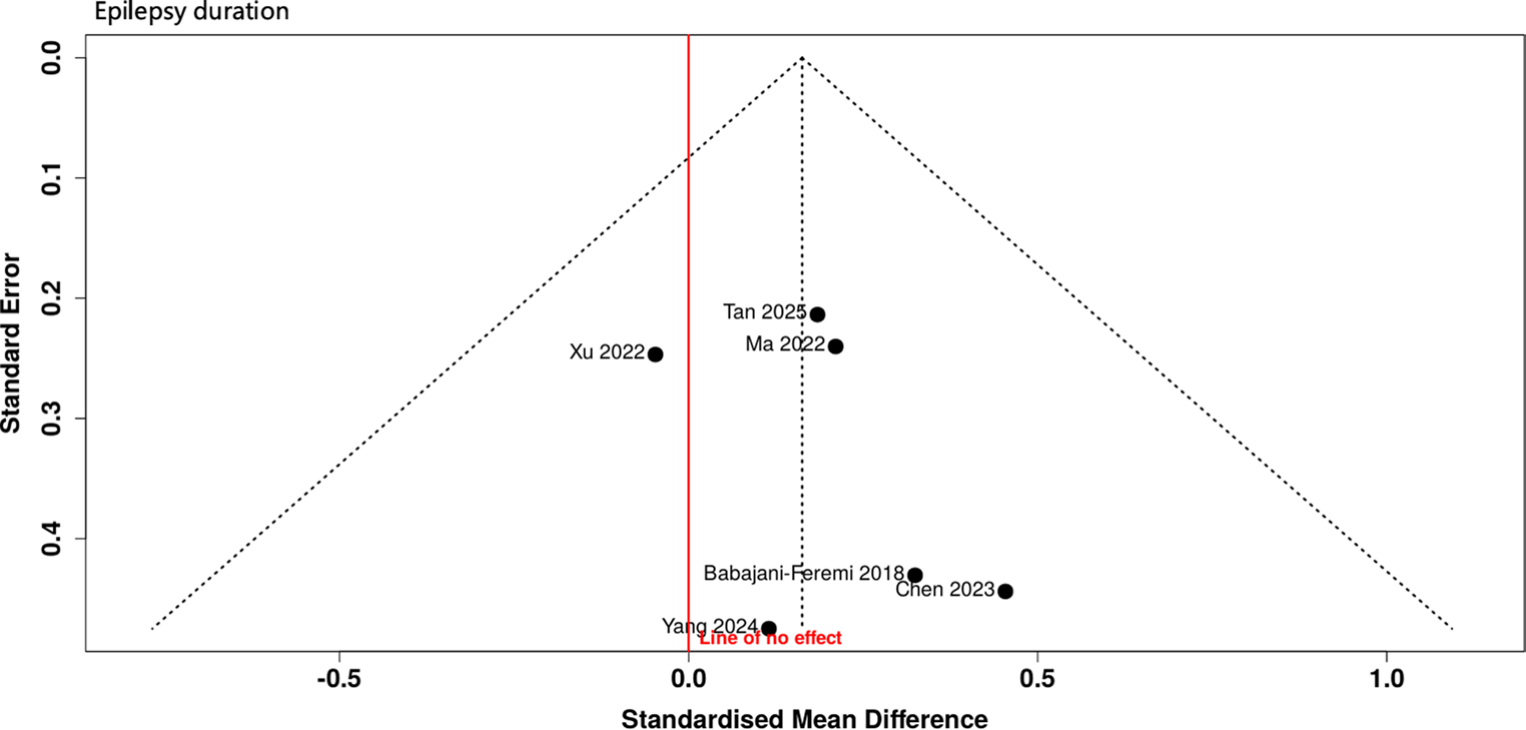
Epilepsy duration funnel plot

A total of 6 studies were investigated with a total of 323 R and 283 NR. Based on the analysis performed using random effects model with inverse variance method to compare the SMD, there is no statistical difference between the two cohorts, the summarized SMD is 0.16 with a 95% CI of -0.07–0.4 (Fig. 9). The test for overall effect does not show a significant effect. We did not find notable variability, implying that the effect sizes across studies were uniform in both size and direction. The funnel plot and Egger tests did not suggest publication bias (Fig. 10).

#### Age at VNS implantation

**Fig. 11:**
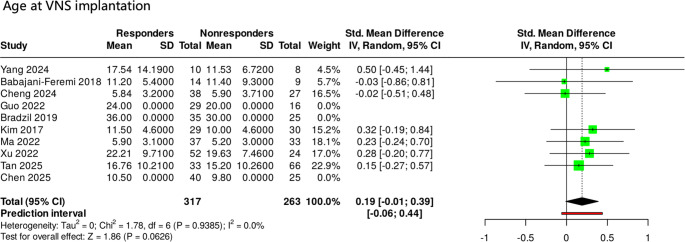
Age at VNS implantation forest plot

**Fig. 12:**
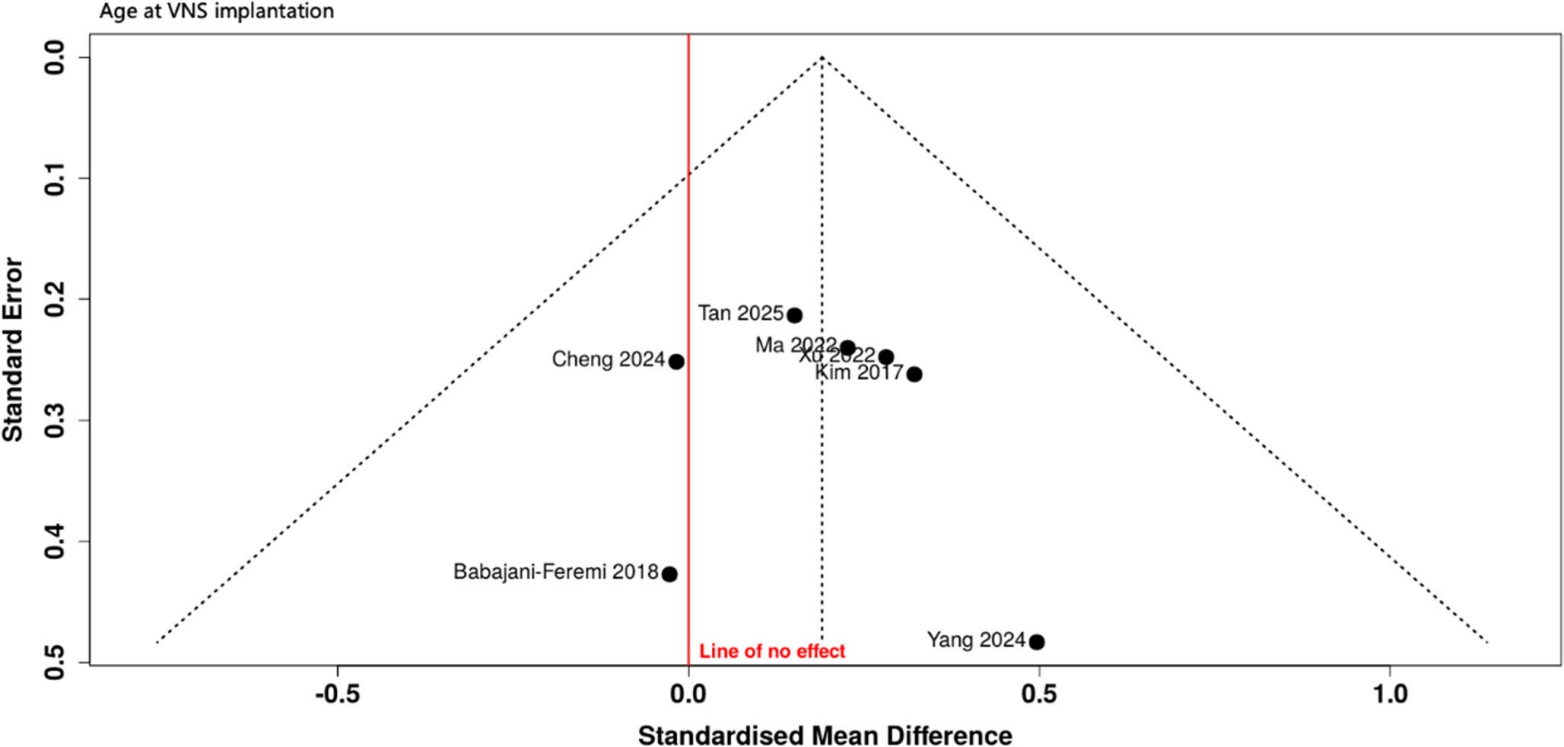
Age at VNS implantation funnel plot

All together 7 cohorts were investigated with a total of 317 R and 263 NR. Based on the analysis performed using random effects model with inverse variance method to compare the SMD, there is no statistical difference between the two cohorts, the summarized SMD is 0.19 with a 95% CI of -0.01–0.39 (Fig. 11). The test for overall effect does not show a significant effect. We did not detect notable variability. The funnel plot and Egger tests did not suggest publication bias (Fig. 12).

#### Focal seizures

**Fig. 13:**
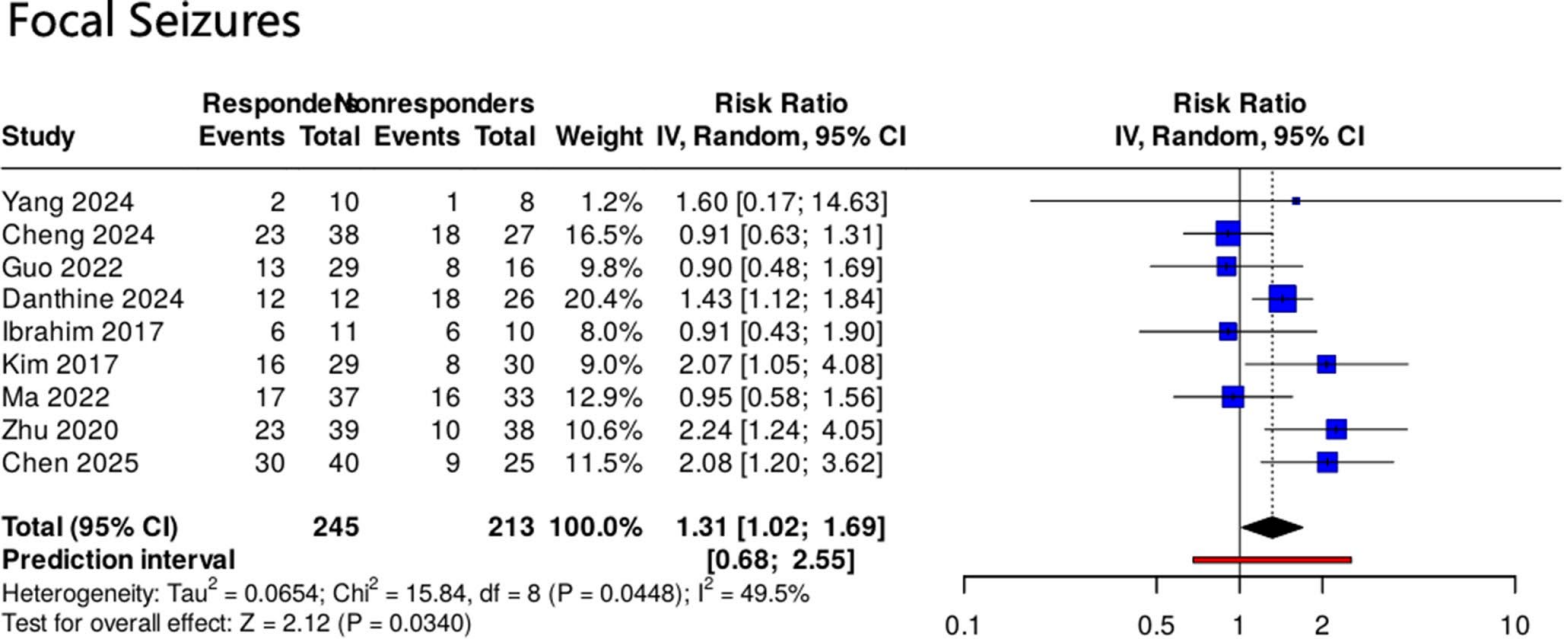
Focal onset of seizures forest plot

**Fig. 14:**
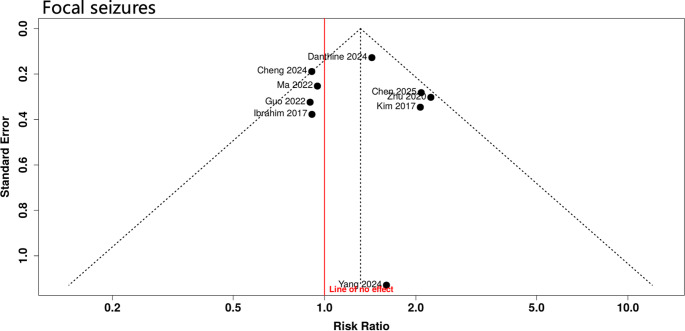
Focal onset of seizures funnel plot

All together 9 studies were analyzed with a total of 245 R and 213 NR. Based on the analysis performed using random effects model with inverse variance method to compare the RR, there is a statistical difference between the two cohorts, the overall RR is 1.31 with a 95% CI of 1.02–1.69. The test for overall effect shows a significance at *p* < 0.05. A significant heterogeneity was detected (*p* = 0.04). The I2 value indicates that 49% of the variability among studies arises from heterogeneity rather than random chance (Fig. 13). The funnel plot and Egger tests did not suggest publication bias (Fig. 14).

## Discussion

Although VNS is established as a safe neuromodulatory option with meaningful benefits on seizure burden and quality of life in selected patients [4–6, 56], treatment outcomes are not uniform: identifying factors that can be considered prognostic of VNS response is therefore crucial. Event though recently a narrative review on this topic was published [57], it did not follow a systematic methodology, limiting reproducibility and increasing the risk of selection bias. Our findings support a multifactorial model of VNS responsiveness in which epilepsy phenotype, electrophysiological markers, and network-level characteristics interact: the literature contains numerous proposed prognostic factors [41], but only a subset appears sufficiently consistent across studies to be clinically applicable. According to the systematic review, earlier intervention appeared to be one of the most reproducible indicator of favorable outcome, and younger age at implantation and shorter epilepsy duration were repeatedly associated with improved seizure control. In the meta-analysis, while focal seizures were significantly associated with response, while earlier age at seizure onset also differentiated R from NR, epilepsy duration and age at implantation did not show significant effects in pooled analyses, likely reflecting heterogeneity between the studies. An important methodological caveat in interpreting the apparent benefit of earlier intervention concerns the potential confounding effect of stimulation duration. Because R rates progressively increase over time following implantation, patients implanted earlier inevitably accumulate longer exposure to neuromodulation, and most retrospective studies do not adequately disentangle these two effects. Studies adopting time-matched analyses or fixed follow-up windows [30, 37] have attempted to control for this bias, but heterogeneity in follow-up duration across the included cohorts limits firm conclusions. Therefore, the association between earlier implantation and better outcome should be interpreted as suggestive rather than definitive, and prospective studies with predefined follow-up endpoints are needed to clarify the independent contribution of timing versus duration of stimulation. Another discrepancy between results of the systematic review and our meta-analysis was detected in evaluating male gender: while individual cohort studies had generally not detected a significant impact of gender on VNS response, we identified a small but statistically significant association of male gender with response (RR 1.14). The modest effect size and the possibility of residual confounding could still limit the clinical relevance of this finding, which should be considered exploratory and warrants confirmation in prospective cohorts with adequate adjustment for potential confounders.

Finally, genetic etiology did not distinguish R from NR, suggesting that current clinical classifications may be too broad to capture gene-level factors highlighted in SNP-focused studies [52].

Electrophysiological studies suggest that EEG and MEG features may provide prognostic information for VNS response; however, reported biomarkers are heterogeneous due to differences in acquisition methods and analytic endpoints, including IED distribution, oscillatory bands, entropy measures, symmetry indices, and event-related potentials [17, 19–21]. This heterogeneity limits cross-study synthesis and makes quantitative analyses infeasible, as already highlighted in earlier work [9] and reinforced by the present review.

Nevertheless, the analyzed data show that focal or multifocal IEDs and focal EEG abnormalities tend to associate with better seizure outcomes after VNS [16, 17], whereas generalized or bilateral patterns and increased interhemispheric asymmetry are more frequent in NR [19, 20]. These findings suggest that focal epileptogenic organization may preserve modulatory pathways through which VNS exerts its network effects. Similarly, measures probing network topology such as MEG graph metrics, support the idea that R differ at the level of large-scale organization [23], consistent with connectivity-based models showing thalamocortical circuitry involvement [24]. These findings should be interpreted with caution in the context of complex electroclinical syndromes such as Lennox–Gastaut syndrome, which are not strictly focal or generalized but rather characterized by diffuse and multifocal network dysfunction with mixed semiological features. In such conditions, the association between focal seizure onset and VNS response may not fully capture the underlying pathophysiology. This further supports the concept that VNS responsiveness is better understood within a network-level framework rather than through a dichotomous classification of focused and generalized epilepsy. The most clinically relevant implication of our findings is that VNS should be considered earlier in carefully selected patients, rather than reserved only as a late-stage salvage therapy. Multiple observational series associate shorter disease duration and earlier implantation with better outcomes [27–31, 33], and at least one study suggests that early partial response predicts long-term benefit [37]. Although epilepsy duration was not statistically significant in our meta-analysis, several cohorts [31(p202), 32, 36]reported increasing responder rates over time, suggesting that VNS benefits may accumulate progressively. Consequently, delaying implantation may postpone potential long-term improvement. In patients who are not candidates for resective surgery, particularly those with focal seizure phenotypes or focal IED patterns, earlier VNS consideration may be warranted to reduce the cumulative burden of ongoing seizures, polytherapy, and healthcare utilization [4, 9].

Predictive modeling studies represent an important evolution from single-variable predictors toward integrated stratification tools. The different studies evaluated share the vision of VNS response as a network phenomenon [24, 42–47] but are mostly retrospective, developed on modest sample sizes, and not externally validated: high accuracy data should therefore be interpreted cautiously. A network-level interpretation offers the most coherent framework for understanding the prognostic factors identified in our review. Studies based on pre-operative MEG-EEG have shown that R have a more organized brain network than NR, with connectivity patterns resembling those of healthy controls suggesting that a sufficiently preserved network architecture is needed for VNS to exert its effect [23]. This view is reinforced by imaging data on thalamocortical circuitry: stronger resting-state connectivity between the thalami and the anterior cingulate and insular cortex [24], together with better-preserved thalamocortical white-matter integrity on DTI [51], identifies the anatomical pathway through which vagal afferents reach the cortex and modulate it. The locus coeruleus–noradrenergic (LC-NE) system is the neurotransmitter pathway most consistently linked to VNS efficacy. Although direct measurement in humans is not feasible, De Taeye et al. showed that VNS increases the amplitude of the P3 event-related potential, which is a non-invasive marker of LC-NE activation, only in R: a > 20% increase, in fact, identified R with 90% specificity [26]. Predictive models combining EEG, imaging, and event-related potential features report accuracies of 80–90%, but rely on small single-centre cohorts with limited external validation, as shown by the failure of pre-operative EEG symmetry to replicate in an independent cohort [19, 20]. Therefore, these models are more useful for defining which multimodal features require standardization and prospective validation rather than providing clinical calculators. New information coming from the analyses of imaging, autonomic, and genetic biomarkers offer potentially important complementary information [49, 50, 52–55]. Imaging based approaches, including perfusion changes on SPECT [50] and structural connectivity integrity on diffusion imaging [51], reinforce the centrality of thalamocortical and limbic circuitry in mediating response. Similarly, autonomic markers such as sleep HRV patterns [55] align with the vagal mechanism of action and may provide accessible physiological stratification tools.

## Limitations

This review is limited by the heterogeneity and overall quality of the available evidence. Most included studies were retrospective, with variable follow-up and heterogeneous patient populations. Outcome definitions and analytical approaches were often inconsistent, limiting comparability, and residual confounding cannot be excluded.

## Conclusions

In the pooled meta-analysis, focal seizure pattern, younger age at epilepsy onset, and male gender emerged as the most consistent prognostic factors of favorable VNS response. Shorter epilepsy duration and earlier age at implantation, although consistently reported as favorable in the qualitative synthesis of the systematic review, did not reach statistical significance in our meta-analysis, likely owing to study heterogeneity, and should therefore be regarded as suggestive rather than confirmed prognostic factors. EEG connectivity findings further support the role of preserved network organization in treatment response. Methodological heterogeneity and the predominance of retrospective studies limit immediate clinical translation; prospective, multicenter studies are needed to develop validated prediction models. 

## Data Availability

No datasets were generated or analysed during the current study.

## Abbreviations

ADK: Adenosine Kinase
CI: Confidence interval
DRE: Drug-Resistant Epilepsy
EEG: Electroencephalography
fMRI: Functional Magnetic Resonance Imaging
IEDs: Interictal Epileptic Discharges
LC-NE: Locus coeruleus–noradrenergic
MEG: Magnetoencephalography
MRI: Magnetic Resonance Imaging
NR: Non responder
P3: Event-Related Potential Component P3
PICO: Population, Intervention, Comparison, Outcomes
PLI: Phase Lag Index
PRISMA: Preferred Reporting Items for Systematic Reviews and Meta-Analyses
R: Responder
RR: Risk ratio
ROI: Region of Interest
SNPs: Single Nucleotide Polymorphisms
SPECT: Single-Photon Emission Computed Tomography
SMD: Standardized mean difference
SVM: Support Vector Machine
VNS: Vagus Nerve Stimulation
VN-EPs: Vagus Nerve Evoked Potentials
wPLI: Weighted Phase Lag Index
PTE: Post-Traumatic Epilepsy
IEDs: Interictal Epileptiform Discharges
rs-MEG: Resting-State MEG
AUC: Area Under the Curve
ROC: Receiver Operating Characteristic
